# Enzyme Immobilisation on Cellulose via Bifunctional Reactive Dyes: A Simple Route to Textile-Based Biocatalysts

**DOI:** 10.3390/polym18101205

**Published:** 2026-05-15

**Authors:** Guigang Shi, Yuhui Li, Wenlong Li, Ruoying Zhu, Ying Sun

**Affiliations:** 1 School of Textile Science and Engineering, Tiangong University, Tianjin 300387, China; 2020010037@tiangong.edu.cn (G.S.); 2431010201@tiangong.edu.cn (Y.L.); 2431010211@tiangong.edu.cn (W.L.); 2 Key Laboratory of Advanced Textile Composites, Ministry of Education, Tiangong University, Tianjin 300387, China

**Keywords:** reactive dye, lysozyme, cotton, enzyme immobilisation, antibacterial textile, cold pad–batch dyeing

## Abstract

The durable enzyme functionalisation of cellulosic fibres is often limited by enzyme deactivation under alkaline processing and insufficient wash resistance. Here, a cold pad–batch (CPB)-compatible route integrates reactive dyeing and lysozyme anchoring by using the commercial bifunctional dye C.I. Reactive Red 195 (MCT/VS) as an interfacial mediator to build a cellulose–dye–lysozyme ternary layer. The dye is first fixed on cotton, and residual electrophilic motifs are proposed to facilitate subsequent coupling with nucleophilic residues in lysozyme. A nine-run, four-factor/three-level orthogonal design was used to identify a practical processing window; under the selected condition, an ELISA-equivalent releasable lysozyme level of 53.8 U/L was achieved together with moderate colour strength (K/S = 6.5). The treated fabrics exhibited 96.5% inhibition against *Escherichia coli* and >99.9% against *Staphylococcus aureus* and retained antibacterial functionality after five ISO 105-C06 laundering cycles. Textile-relevant properties were preserved, including colour fastness (Grade 4–5), tensile strength retention (~86%), and capillary wicking close to pristine cotton. This dye-mediated strategy offers a practical route to wash-resistant bioactive interfaces on cellulose fibres and is extendable to regenerated cellulose and wood-pulp-derived cellulosics.

## 1. Introduction

Cotton is widely used in apparel and skin-contact textiles because of its softness and moisture management [1]. Its porous, moisture-retentive structure can also favour microbial colonisation on worn garments, particularly in warm and humid environments. Common isolates reported on cotton include *Staphylococcus aureus* and *Escherichia coli* [2]. Such contamination may contribute to malodour, accelerate textile deterioration, and raise hygiene concerns in medical and skin-contact scenarios [3]. A key requirement for cellulosic substrates is to deliver antibacterial performance that remains effective under real wear and repeated laundering [4].

A range of antibacterial finishes has been investigated for cotton, including inorganic nanoparticles and quaternary ammonium compounds [5,6]. Their use can be limited by biocompatibility concerns and environmental persistence [6,7,8]. Enzyme-based finishes offer a biocompatible alternative for skin-contact applications. Lysozyme is of particular interest because it hydrolyses bacterial peptidoglycan and is generally considered suitable for such uses [9]. The main challenge is durability: physically adsorbed enzymes are readily removed during use and washing, while covalent immobilisation commonly requires substrate preactivation and/or additional coupling reagents that can reduce enzyme activity or leave residual reagents [10,11]. Primary studies on cellulose-based substrates have shown that the covalent anchoring of lysozyme (e.g., via carbodiimide-mediated amide formation on activated cotton/cellulose) can enhance retention and functional performance compared with adsorption, albeit at the cost of additional chemical steps and process complexity [12,13]. At the same time, unambiguous bond-level discrimination on fibre surfaces is challenging using spectroscopy alone; therefore, sequence-dependent controls and wash-relevant behaviour are often used as converging evidence when assessing durable enzyme–cellulose interfaces [14]. A coupling strategy that is both durable and compatible with practical fibre processing conditions is still needed.

Reactive dyes are designed to form covalent bonds with cellulose hydroxyl groups during alkaline fixation [15,16], which makes dye fixation chemistry relevant to surface functionalisation as well as coloration. In this work, C.I. Reactive Red 195 was employed as a colourant and as a molecular mediator. After fixation on cellulose, its electrophilic reactive groups may provide coupling sites for nucleophilic residues in lysozyme, enabling the formation of a cellulose–dye–enzyme interfacial structure [15,17]. Reactive Red 195 contains both monochlorotriazine (MCT) and vinyl sulfone (VS) moieties, offering multiple reaction pathways that may support interfacial coupling under comparatively mild conditions [15,16]. This approach offers a practical route to construct protein-associated interfacial layers on cellulose fibre surfaces.

**Scheme 1:**
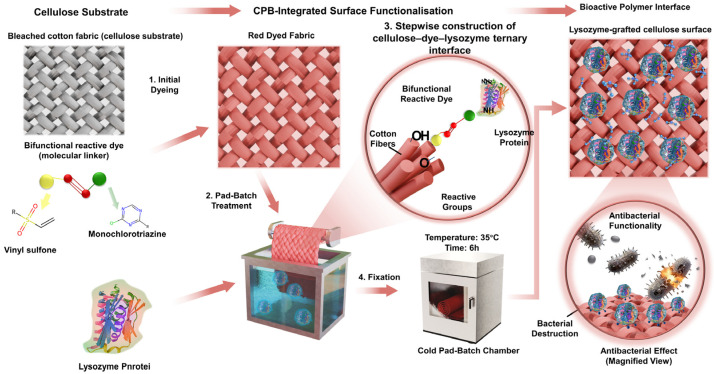
**The CPB-compatible dye-bridged immobilisation of lysozyme on cotton.** C.I. Reactive Red 195 first reacts with cellulose during dyeing and retains residual reactive sites that may enable subsequent coupling with lysozyme, forming a cellulose–dye–lysozyme ternary structure.

Here, we developed a cold pad–batch (CPB) process in which C.I. Reactive Red 195 was used not only as a dye but also as an interfacial mediator for introducing lysozyme onto cotton (Scheme 1). This study was built around a practical question: whether the order of dye fixation and enzyme addition could improve lysozyme retention while maintaining antibacterial activity and basic textile performance. We therefore compared different lysozyme introduction routes, examined the treated fibre surface, and evaluated antibacterial activity, laundering durability, colour performance, mechanical strength, and wettability. In this way, this work explores whether an established reactive dyeing workflow can be adapted to produce a wash-resistant bioactive cotton surface without adding a separate crosslinking step [18].

## 2. Materials and Methods

### 2.1. Materials and Reagents

Desized and bleached plain-woven cotton fabric (180 g/m^2^; warp/weft density: 108 × 58 ends/inch; yarn count: 21 s × 21 s) was supplied by Shandong Weileike Textile and Garment Co., Ltd. (Linyi, China). The commercial heterobifunctional reactive dye C.I. Reactive Red 195 was purchased from Shanghai Anoky Group Co., Ltd. (Shanghai, China) and used as received. Lysozyme (EC 3.2.1.17, from chicken egg white; activity 20,000 U/mg) was obtained from Sigma-Aldrich (St. Louis, MO, USA). The lysozyme sandwich ELISA kit used to quantify the amount of lysozyme was supplied by Shanghai MLBIO Biotechnology Co., Ltd. (Shanghai, China).

### 2.2. Computational Methodology (DFT)

Density Functional Theory (DFT) calculations were performed using the Gaussian 16 software package. The B3LYP functional with D3BJ dispersion correction was used to account for non-covalent interactions. Geometry optimisations and vibrational frequency analyses were carried out using the 6-31G(d,p) basis set for all atoms. Single-point energy calculations were further performed at the B3LYP-D3BJ/6-311+G(d,p) level. Condensed Fukui functions were obtained using the Multiwfn package, and electrostatic potential (ESP) maps were visualised with VMD.

### 2.3. Preparation of Functionalised Cotton via CPB Process

Lysozyme was incorporated into a cold pad–batch (CPB) reactive dyeing workflow to construct a dye-mediated cellulose–enzyme interfacial system. Three introduction sequences were evaluated. Unless stated otherwise, lysozyme was used at 2 g/L, and wet pick-up was maintained at 80%.

Non-dye-coupled lysozyme. To provide a dye-free reference, pristine cotton was padded with the lysozyme solution (2 g/L, pH 8.0) to 80% wet pick-up, followed by the same batching and washing procedure as described below.

Strategy I: Simultaneous introduction (one-bath). Lysozyme (2 g/L) was added directly to the dye liquor containing C.I. Reactive Red 195 (20 g/L), NaCl (10 g/L), and Na_2_CO_3_ (30 g/L; pH ≈ 10). The fabric was padded once with the mixed bath to reach 80% wet pick-up.

Strategy II: Sequential impregnation. The fabric was first padded with the dye bath composed of C.I. Reactive Red 195 (20 g/L), NaCl (10 g/L), and Na_2_CO_3_ (Factor A in the orthogonal design; typically 30 g/L). Without intermediate drying, a second padding was performed using the lysozyme solution (2 g/L), prepared in deionised water and adjusted to pH 8.0. This mildly alkaline condition helped maintain enzyme stability during impregnation, while the alkali retained in the fabric from the first padding step provided the fixation environment required during subsequent batching.

Strategy III: Post-fixation introduction. The fabric was dyed first using the standard CPB procedure (Reactive Red 195/NaCl/Na_2_CO_3_), then rinsed thoroughly to remove unfixed dye. The dyed fabric was subsequently re-padded with the lysozyme solution (2 g/L, pH 8.0).

After padding, samples were wrapped with plastic film to minimise moisture loss and batched at 35 °C to complete fixation. For the route comparison experiments, the batching time was fixed at 10 h for consistency. For the optimised ternary system used in subsequent characterisation, the batching time followed the orthogonal design results (Section 3.2). After batching, all fabrics were rinsed thoroughly with deionised water until the wash liquor became clear, air-dried at room temperature, and stored in sealed bags prior to testing. Based on the optimisation results in Section 3.2, Strategy II was selected to prepare the cellulose–dye–lysozyme ternary system.

### 2.4. Surface Characterisation

The surface properties and chemical composition of the fabrics were analysed using the following techniques:

FTIR-ATR Spectroscopy: The chemical structures of the control and functionalised fabrics were examined using a Fourier Transform Infrared (FTIR) spectrometer (Nicolet iS50, Thermo Fisher Scientific, Waltham, MA, USA), equipped with an Attenuated Total Reflection (ATR) accessory. Spectra were recorded within the range of 4000–600 cm^−1^ at a resolution of 4 cm^−1^.

XPS Analysis: Surface elemental composition and bonding states were determined via X-ray Photoelectron Spectroscopy (XPS) using a Thermo Fisher ESCALAB 250Xi system with monochromatic Al Kα radiation (ESCALAB 250Xi, Thermo Fisher Scientific, Waltham, MA, USA). Binding energies were calibrated using the C1s adventitious peak at 284.8 eV for accuracy.

Scanning Electron Microscopy (SEM): The surface and cross-sectional morphologies of the fibres were examined using a field-emission scanning electron microscope (S-4800, Hitachi High-Tech Corporation, Tokyo, Japan) operated at an accelerating voltage of 5.0 kV. Fabric specimens were dried, mounted on aluminium stubs using conductive carbon tape, and sputter-coated with a thin gold layer prior to imaging. For cross-sectional observation, samples were cryo-fractured after liquid nitrogen cooling to expose the internal structure, and the fractured surfaces were imaged under the same conditions.

X-ray Diffraction (XRD): The crystalline structure of cellulose was examined using an X-ray diffractometer equipped with a Cu Kα radiation source (D8 Advance, Bruker AXS GmbH, Karlsruhe, Germany). The diffractograms were used to assess whether the characteristic Cellulose I diffraction pattern was retained after CPB dyeing and lysozyme introduction.

### 2.5. Lysozyme Quantification (Extraction-Based Sandwich ELISA)

Releasable lysozyme was quantified from the physiological saline extracts of the treated fabrics using a commercial Lysozyme (LZM) sandwich ELISA kit (Shanghai MLBIO Biotechnology Co., Ltd., Shanghai, China). The ELISA response is reported as the ELISA-equivalent lysozyme level (U/L, as defined by the kit) and was used as a consistent indicator to compare enzyme-associated levels among different fabrication routes.

For extraction, fabric specimens were cut into small pieces and incubated in physiological saline (0.9 wt% NaCl) at a fixed liquor ratio of 1:50 (0.1 g fabric in 5 mL saline) for 24 h at 37 °C with gentle shaking (120 rpm). The extract was collected, and the supernatant was used for ELISA analysis. A standard series (0, 25, 50, 100, 200, and 400 U/L) was prepared by the serial dilution of the lysozyme standard provided with the kit.

For the assay, 50 μL of each standard solution was added to the antibody-coated wells. For samples, 10 μL of fabric extract was mixed with 40 μL of the kit-supplied sample diluent (5-fold dilution), followed by the addition of 100 μL of HRP-conjugated detection antibody. After incubation at 37 °C for 60 min, the plate was washed five times. Substrate solutions A and B (50 μL each) were then added and allowed to develop for 15 min in the dark at 37 °C. The reaction was stopped, The reaction was stopped, and absorbance was measured at 450 nm using a microplate reader (Infinite F50, Tecan Group Ltd., Männedorf, Switzerland). Lysozyme levels were calculated from the standard curve and corrected using the dilution factor (×5).

### 2.6. Antibacterial Activity Assay (Dynamic Shake Flask Method)

#### 2.6.1. Bacterial Culture and Inoculum Preparation

Cryopreserved bacterial stocks of *Escherichia coli* (*E. coli*, ATCC 25922) and *Staphylococcus aureus* (*S. aureus*, ATCC 6538) were thawed and streaked onto nutrient agar plates for activation. Single colonies were inoculated into nutrient broth and incubated at 37 °C. After reaching the logarithmic growth phase, bacterial cells were harvested by centrifugation (4000 rpm, 10 min), resuspended in sterile PBS, and adjusted to a final concentration of 1 × 10^6^–1 × 10^7^ CFU/mL.

#### 2.6.2. Sample Sterilisation

Prior to testing, fabric samples were sterilised by UV irradiation (2 h) to eliminate any pre-existing microbial contamination.

#### 2.6.3. Antibacterial Test Procedure

Sterilised fabric samples were immersed in flasks containing fresh nutrient broth inoculated with 1% (*v*/*v*) bacterial suspension. The flasks were incubated at 37 °C with shaking at 180 rpm for 24 h. After incubation, the suspension was serially diluted with sterile PBS, plated on nutrient agar, and incubated at 37 °C for 24 h. Colony-forming units (CFUs) were counted.

#### 2.6.4. Calculation

The antibacterial reduction rate was calculated using the following formula:

$$R\;\left(\%\right)=\frac{A-B}{A}\;\times 100\%$$

where A is the colony count (CFU/mL) of the control flask (pristine cotton), and B is the colony count (CFU/mL) of the test flask (functionalised fabric).

### 2.7. Evaluation of Dyeing and Physical Properties

The dyeing performance and physical durability of the treated fabrics were assessed using standard industrial protocols:

Colour Strength (K/S): Colour yield was quantified by measuring the K/S value using a Datacolor spectrophotometer under Illuminant D65 with a 10° standard observer. The K/S values were calculated using the Kubelka–Munk equation.

Colour Fastness: The durability of dyeing against washing and rubbing (dry and wet) was evaluated according to ISO 105-C06 and ISO 105-X12 standards, respectively.

Wash Durability Test: The laundering durability of antibacterial functionality was evaluated according to ISO 105-C06 (GB/T 3921-2008). The samples were washed in a standard soap solution at 40 °C for 30 min per cycle. This procedure was repeated for five consecutive cycles. After each cycle, the fabrics were thoroughly rinsed with deionised water and air-dried before the subsequent test or the next laundering cycle.

Tensile Strength: The mechanical integrity of the fabrics was assessed by measuring the breaking strength in both the warp and weft directions using a Universal Testing Machine (Instron 3369, Instron Corporation/825 University Ave, Norwood, MA, USA), following the ASTM D5035 method.

Wettability (Capillary Wicking): The wettability of the fabrics was assessed by the capillary rise method according to FZ/T 01071-1999. Fabric strips (25 cm × 3 cm) were suspended vertically with their lower ends immersed in distilled water. The capillary wicking height was recorded after 30 min. Measurements were conducted in triplicate for each sample, and the average values were reported.

## 3. Results

### 3.1. Process Window Screening via L9 (3^4^) Orthogonal Design

To define a workable CPB window for dye-mediated lysozyme immobilisation, an L9 (3^4^) orthogonal design was used by varying alkali concentration (A), batching time (B), dye concentration (C), and lysozyme dosage (D). All runs were carried out using the sequential impregnation route (Strategy II) to maintain a consistent enzyme introduction sequence. Lysozyme-associated levels were evaluated using the ELISA-equivalent releasable lysozyme signal obtained from physiological saline extracts, which was used as a practical indicator for comparing different processing conditions. Antibacterial performance against *S. aureus* and colour strength (K/S) were recorded as functional and dyeing-related responses (Table 1). It should be noted that batching time was fixed at 10 h only for the route comparison experiments (Strategy I–III), whereas in orthogonal screening, it was treated as an independent factor (6–14 h) to establish the practical CPB operating window.

The range analysis summarised in Table 2 shows that lysozyme dosage (D) exerted the strongest influence on the releasable ELISA level (R = 21.0), followed by alkali concentration (A, R = 13.6). In comparison, batching time (B, R = 3.8) and dye concentration (C, R = 3.1) contributed less within the tested range. A similar pattern was observed for antibacterial activity against *S. aureus*, where D produced the largest variation (R = 28.4), and A remained the second most influential factor (R = 22.7). By contrast, colour strength was mainly governed by dye concentration (R = 4.63), while lysozyme dosage had only a minor effect on K/S (R = 0.43). Here, K values represent the average response at each factor level, and the separation of dominant factors suggests that shade depth can be tuned through dye concentration without strongly disturbing the enzyme-associated response under CPB conditions.

Among the nine experimental runs, A2B1C2D3 (30 g/L alkali, 6 h batching, 20 g/L dye, and 4 g/L lysozyme) produced the highest ELISA-equivalent lysozyme level (53.8 U/L) together with the highest antibacterial rate (99.9%) while maintaining moderate colour strength (K/S = 6.5). This condition was therefore selected as the optimised setting for the subsequent mechanistic verification and performance evaluation of the cellulose–dye–lysozyme ternary interfacial system.

With the processing window established, the next step was to clarify how Reactive Red 195 enables interfacial coupling between cellulose hydroxyl groups and nucleophilic residues in lysozyme through its electrophilic reactive domains (VS/MCT). Conceptual DFT calculations were therefore performed to locate electrophilic sites on the dye that are susceptible to nucleophilic attack, providing molecular-level support for the proposed dye-bridging mechanism discussed in the following section [19].

### 3.2. DFT-Guided Rationale for Dye-Mediated Coupling

**Figure 1:**
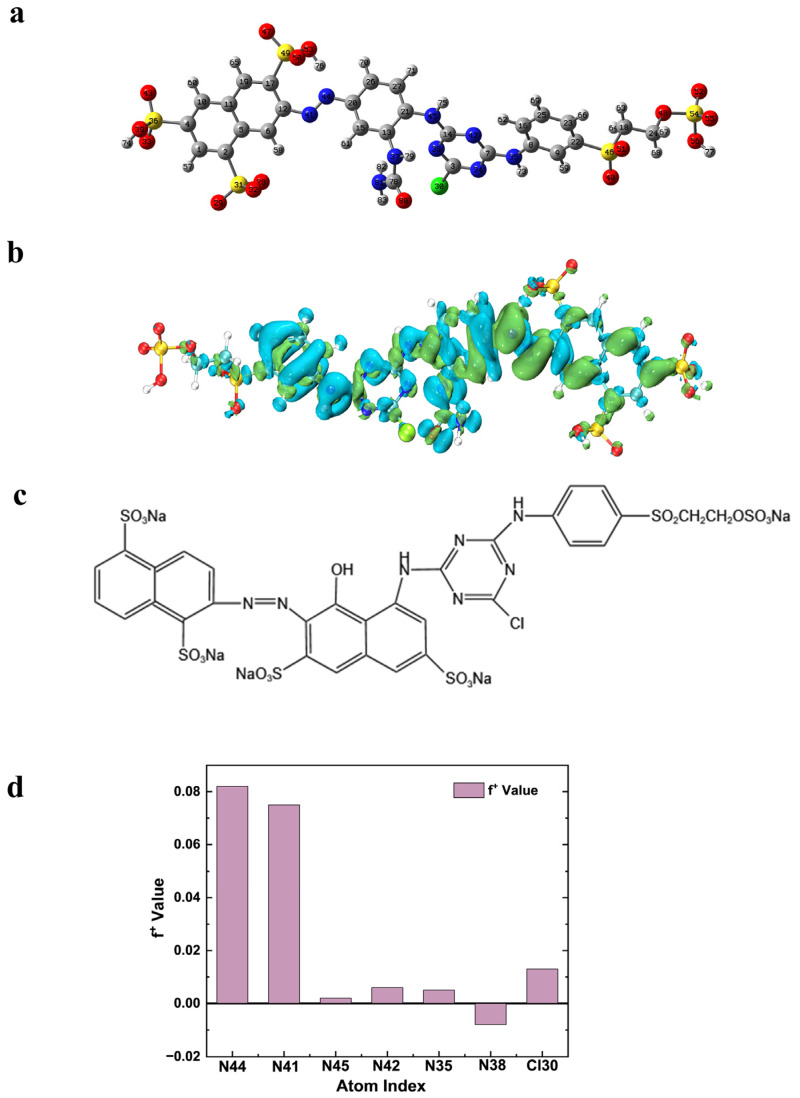
**DFT-based electronic reactivity analysis using an RR195-derived computational model.** (**a**) The optimised three-dimensional computational model used for the DFT analysis. (**b**) The Dual Descriptor (DD) isosurface of the optimised model, showing the spatial distribution of the local electrophilic/nucleophilic character. (**c**) The reference two-dimensional chemical structure of C.I. Reactive Red 195 (RR195). (**d**) Condensed Fukui f^+^ values for representative atoms in the analysed reactive domain. Full model definition, atom numbering, and detailed computational outputs are provided in the Supporting Information (Section S4, Table S4, and Figures S2 and S3).

For the cellulose–dye–lysozyme ternary system, the key question is whether the dye retains an electronically activated region accessible to both cellulose hydroxyls and lysozyme amino groups. To examine this, DFT calculations were carried out on an RR195-derived model centred on the reactive domain of the dye. The full two-dimensional structure of RR195 is shown separately in Figure 1c for chemical reference, whereas the descriptors discussed below were obtained from the derived model.

RR195 is a heterobifunctional reactive dye containing a monochlorotriazine (MCT) group and a vinyl sulfone (VS) group, the latter generated from the sulphatoethylsulfone precursor under alkaline conditions. The electronic features relevant to nucleophilic attack were evaluated using the Dual Descriptor (DD) and the condensed Fukui function (f^+^).

The DD isosurface indicates that the strongest electrophilic character is concentrated in the MCT/VS part of the model rather than being distributed over the chromophoric framework as a whole (Figure 1b). The condensed Fukui results show the same trend. N44 and N41 give the highest f^+^ values, 0.0823 and 0.0748, respectively (Figure 1d). These atoms are not assigned here as the actual bond-forming sites but are taken as markers of the most electron-deficient part of the reactive domain. This pattern is consistent with the enhanced electrophilic character near the carbon bearing the leaving group in the MCT ring and near the electrophilic site associated with the VS functionality.

In this study, DFT analysis is used mainly as supporting chemical evidence for the proposed dye-mediated process. The result suggests that once fixed on cotton, RR195 may still provide a locally activated environment for subsequent interaction with lysozyme nucleophiles under the CPB conditions used here. Any specific bonding interpretation should therefore be considered together with the experimental results presented below.

### 3.3. Effect of Incorporation Route on ELISA Response

**Figure 2:**
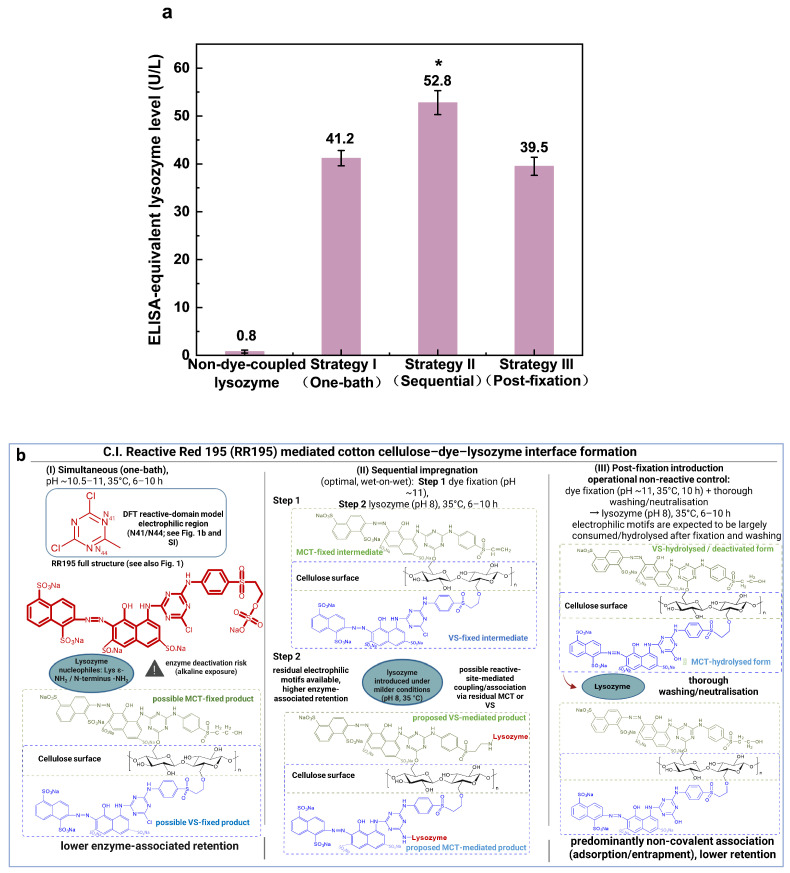
Optimisation of lysozyme incorporation in CPB process. (**a**) ELISA-equivalent lysozyme level (U/L) measured in physiological saline extracts of cotton fabrics prepared by different introduction routes: non-dye-coupled lysozyme, Strategy I (one-bath), Strategy II (sequential), and Strategy III (post-fixation). Data are presented as mean ± SD (*n* = 3), *p* < 0.05 compared with Strategy I and Strategy III. (**b**) Proposed RR195-mediated pathways for cellulose–dye–lysozyme interfacial structure formation under three introduction strategies.

The extracted ELISA-equivalent level depended strongly on the stage at which lysozyme was introduced into the CPB process. Without RR195, only a background signal was obtained (0.8 U/L), indicating that lysozyme was barely retained on cotton in the dye-free reference. Once the dye was included, the detected level increased markedly in all three routes. The highest value was obtained for Strategy II (52.8 U/L), whereas Strategy I and Strategy III gave lower values of 41.2 and 39.5 U/L, respectively (Figure 2a).

For Strategy I, lysozyme remained in the bath during the alkaline fixation stage required for reactive dyeing. Several hours under strongly alkaline conditions would be expected to impair protein stability, which could reduce the amount later recovered by ELISA. Strategy III removed this alkaline burden, but lysozyme was added only after dye fixation, washing, and neutralisation. By then, the original reactive motifs of the dye were likely to have been largely consumed by cellulose fixation or lost through hydrolysis. Under these conditions, the retained enzyme is more likely to come from adsorption and physical entrapment than from a continuing dye-mediated reaction.

In Strategy II, RR195 was first allowed to react with cellulose, and lysozyme was introduced afterwards in a second wet-on-wet step under milder conditions (pH 8, 35 °C). The enzyme was therefore not exposed to the harsh fixation stage but still came into contact with a dye-modified fibre surface before all reactive character had disappeared. This probably accounts for the higher lysozyme level observed for Strategy II.

Figure 2b summarises the chemical logic behind these three routes. RR195 may be fixed to cellulose through either the MCT route or the VS route, giving corresponding dye-fixed interfacial species. When lysozyme is added in Strategy II, the fibre surface may still retain some dye-derived electrophilic character, so subsequent interaction, and possibly coupling, with lysozyme nucleophiles remains chemically plausible. In Strategy III, the dye skeleton remains on the fibre, but most of the reactive motifs are expected to have been deactivated before enzyme addition. Strategy III was therefore used here mainly as a practical non-reactive reference. This reading also agrees with the DFT results in Figure 1, which place the main electrophilic character in the RR195 reactive domain.

The ELISA data here should not be over-interpreted. The values in Figure 2a were obtained from physiological saline extracts, so they represent extraction-based ELISA-equivalent levels rather than direct measurements of catalytic activity on the fibre surface. ELISA alone also cannot show whether the retained lysozyme is covalently attached or held by strong non-covalent interactions. Any bonding interpretation must therefore be considered together with the later structural and functional results.

### 3.4. Surface Characterisation of Ternary System

**Figure 3:**
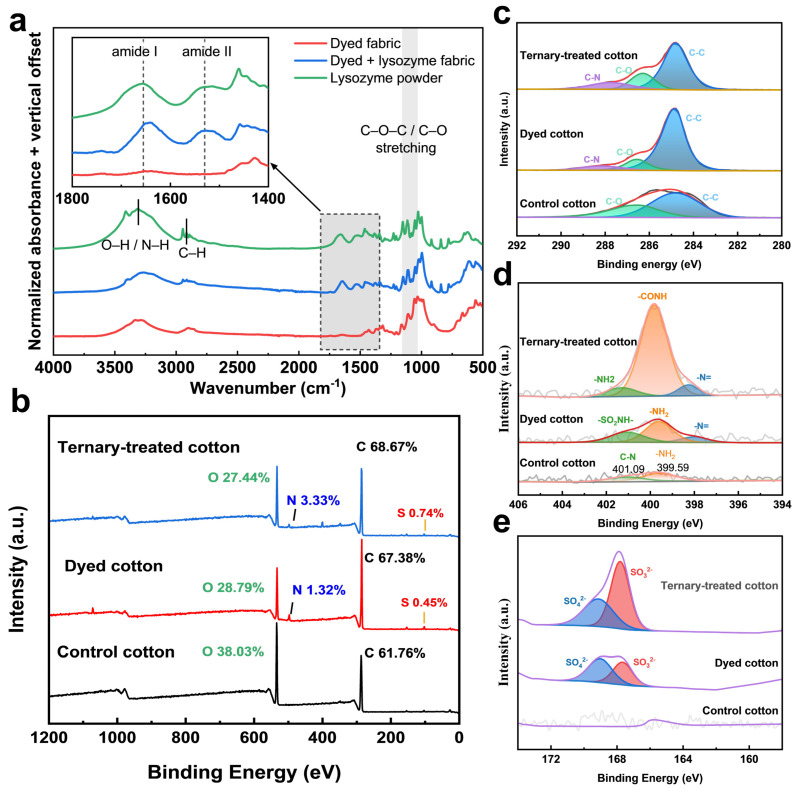
FTIR-ATR and XPS analysis of treated cotton samples. (**a**) FTIR-ATR spectra of dyed cotton, ternary-treated cotton, and lysozyme powder (inset: 1800–1400 cm^−1^). (**b**) XPS survey spectra of control cotton, dyed cotton, and ternary-treated cotton, together with surface atomic composition. (**c**–**e**) High-resolution XPS spectra of C 1s, N 1s, and S 2p regions.

Figure 3a shows that the spectra of dyed cotton and ternary-treated cotton were still dominated by the characteristic bands of cellulose, with no major change in the overall profile after the CPB treatment. This suggests that the cellulose backbone remained intact. Lysozyme powder was included as a reference and displayed the expected amide I and amide II bands in the 1800–1400 cm^−1^ region. Against the dyed cotton spectrum, the ternary-treated sample showed only slight but reproducible changes in this window, mainly as weak shoulders and small baseline disturbances near the amide-related region. These changes are subtle and are not sufficient by themselves to establish a definite bonding mode at the fibre surface. Even so, when viewed together with the lysozyme reference spectrum and the XPS results, they are consistent with the presence of protein-related surface functionalities in the ternary-treated sample.

XPS further resolves the elemental evolution at the outermost surface. The survey spectra (Figure 3b) show the expected C and O signals for all samples, while N and S increase stepwise across the three conditions. Control cotton contained negligible N and no detectable S, as expected for unmodified cellulose. After dyeing, both N and S became measurable (N 1.32 at.% and S 0.45 at.%), reflecting the introduction of the RR195 layer bearing triazine nitrogen and sulfonate groups. Following lysozyme introduction, N increased to 3.33 at.% and S to 0.74 at.%, giving a consistent trend of Control < Dyed < Ternary for both elements. This stepwise enrichment supports a sequential surface modification process, in which the dye layer persists and the subsequent protein step adds additional nitrogen-containing functionality at the interface.

In the high-resolution C 1s region (Figure 3c), the spectra remain dominated by cellulose-derived carbon environments (C–C/C–H and C–O), with only moderate changes in the relative contributions of oxygen- and nitrogen-associated components after dyeing and ternary treatment. This behaviour is consistent with a surface-limited modification that leaves the bulk cellulose chemistry largely intact. In the N 1s region (Figure 3d), the dyed sample shows dye-related nitrogen environments, whereas the ternary-treated cotton exhibits a more pronounced amide-like contribution (assignable to –CONH–) together with amine-type nitrogen. The increased N 1s intensity aligns with the higher survey-level N content, indicating that the additional nitrogen originates from the protein-associated overlayer.

The S 2p spectra (Figure 3e) capture the sulphur chemical state. No S 2p signal is detected for control cotton. Dyed cotton displays the expected sulfonate-related doublet, and the ternary-treated cotton preserves the same sulfonate signature with higher overall intensity, indicating that dye-derived sulphur remains chemically stable through the protein introduction step. Taken together, the amide features in FTIR-ATR and the stepwise N/S enrichment in XPS provide converging evidence that RR195 acts as a persistent interfacial layer and enables the formation of a lysozyme-associated, nitrogen-enriched outer layer, supporting the construction of the ternary surface structure.

**Figure 4:**
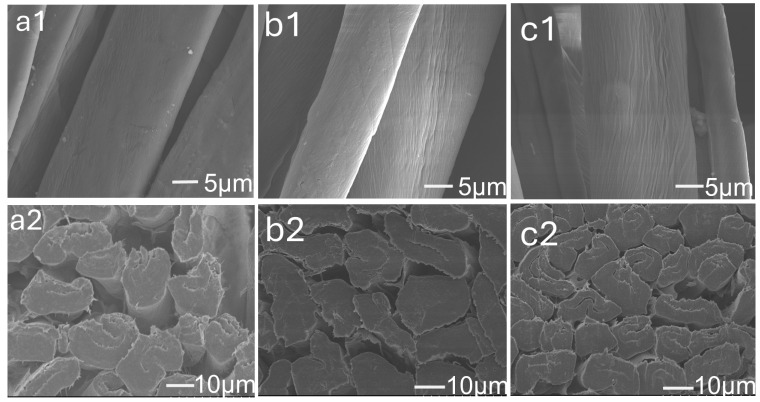
SEM observations of surface morphology and apparent cross-sectional compactness of (**a**) control cotton, (**b**) dyed cotton, and (**c**) ternary-treated cotton. Scale bars: 5 μm for surface images (**a1**–**c1**) and 10 μm for cross-sectional images (**a2**–**c2**).

Figure 4 compares the surface and cross-sectional morphologies of the control cotton, dyed cotton, and ternary-treated cotton. In the surface images, the three samples retain the typical longitudinal features of cotton fibres. No obvious cracking, etching, fibrillation, or surface collapse can be seen after CPB dyeing or after lysozyme introduction, suggesting that the treatment did not visibly damage the fibre surface.

### 3.5. Fibre Morphology and Apparent Fibre Bundle Compactness

The cross-sectional images show a somewhat different fibre bundle arrangement. The control sample contains more visible inter-fibre spaces, whereas the dyed and ternary-treated samples appear more closely packed. This change is most evident in the ternary-treated cotton, where the gaps between neighbouring fibres are less pronounced. Such a morphology is consistent with a tighter fibre bundle arrangement after dye fixation and lysozyme introduction. However, the cross-sectional appearance of woven cotton can also be influenced by local yarn packing, fibre orientation, and the fracture plane during sample preparation. Therefore, this result should be regarded as a morphological indication of increased apparent compactness, rather than direct proof of bulk cellulose densification or inter-chain crosslinking.

### 3.6. Antibacterial Performance

#### 3.6.1. Activity Against *E. coli* and *S. aureus*

**Figure 5:**
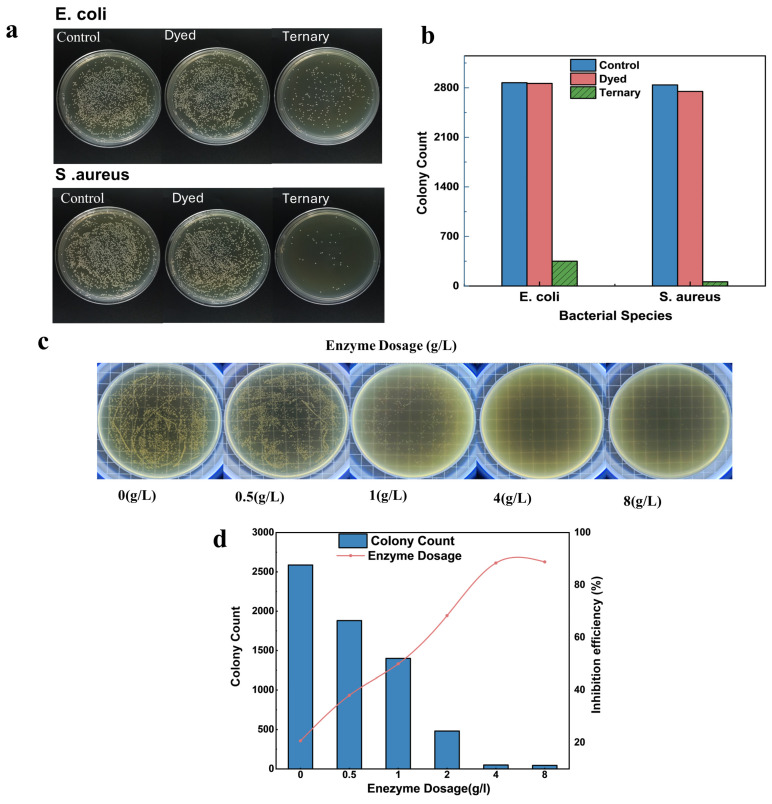
Antibacterial performance of functionalised cotton fabrics. (**a**) Representative agar plates and (**b**) colony counts for *E. coli* and *S. aureus* after contact with control, dyed, and ternary fabrics. (**c**) Agar plates and (**d**) dose–response results of ternary fabrics prepared with different lysozyme dosages.

The antibacterial performance of the functionalised fabrics was assessed against *E. coli* and *S. aureus* by agar plate observation and viable colony counting. As shown in Figure 5a, both the control and dyed samples exhibited dense bacterial growth, indicating that pristine cotton and Reactive Red 195 alone provided negligible inhibition under the present conditions. In contrast, the ternary fabric resulted in a pronounced decrease in colony formation for both strains, confirming that antibacterial activity was introduced upon lysozyme immobilisation.

The quantitative results in Figure 5b support these observations and further show a stronger reduction for *S. aureus* than for *E. coli*. This behaviour is consistent with differences in cell envelope structure: lysozyme hydrolyses peptidoglycan, which is directly exposed in Gram-positive bacteria, whereas Gram-negative bacteria are protected by an outer membrane that can limit enzyme access.

#### 3.6.2. Dose-Dependent Optimisation

To determine a practical lysozyme loading, ternary fabrics were prepared using lysozyme concentrations of 0, 0.5, 1, 4, and 8 g/L and evaluated for antibacterial performance. As shown in Figure 5c, bacterial growth on agar plates decreased progressively with increasing lysozyme dosage. The corresponding colony counts (Figure 5d) declined sharply as the dosage increased from 0 to 4 g/L.

When the dosage was further increased to 8 g/L, only a marginal additional improvement was observed, indicating diminishing returns under the current CPB conditions. Considering both efficacy and processing practicality, 4 g/L was selected as the working dosage for subsequent experiments. Since laundering resistance is essential for practical textile applications, the durability of the antibacterial effect was further examined by washing cycle tests.

### 3.7. Impact on Textile Properties

To assess the practical applicability of the functionalised fabrics, the influence of the optimised lysozyme immobilisation process (Strategy II) on key textile properties was evaluated, including colour performance, mechanical strength, crystalline structure, and wettability.

**Figure 6:**
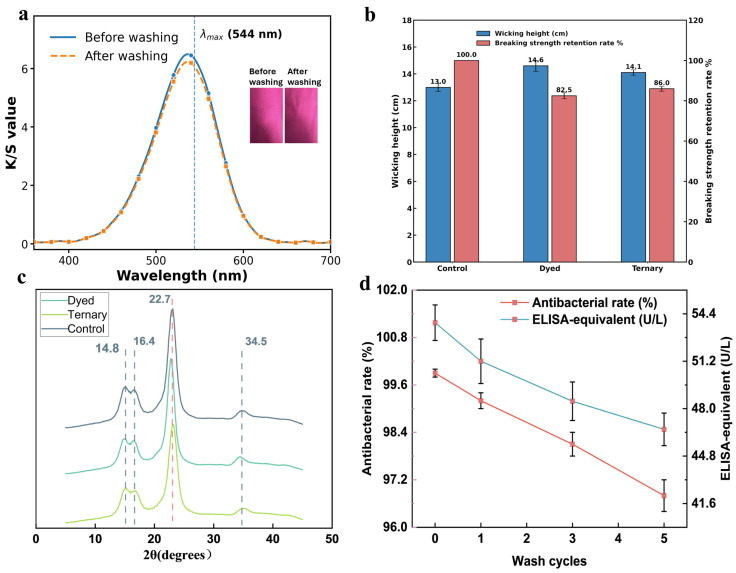
Washing durability and structural characterisation of ternary-treated cotton fabrics. (**a**) Kubelka–Munk (K/S) spectra of ternary fabrics before and after washing, with maximum absorption wavelength (λ_max) indicated; insets show corresponding fabric appearances. (**b**) Wicking height and breaking strength retention of control, dyed, and ternary fabrics. (**c**) X-ray diffraction patterns of control, dyed, and ternary cotton fabrics. (**d**) Antibacterial rate and ELISA-equivalent lysozyme content of ternary fabrics as function of washing cycles.

Colour yield and fastness are prerequisite properties for functional cotton, particularly when bioactivity is introduced. The Kubelka–Munk spectra (Figure 6a) show that the ternary-treated fabric has a K/S comparable to the dyed control before washing, indicating that lysozyme immobilisation does not reduce the colour yield of Reactive Red 195. After repeated laundering, only a slight decrease in K/S is observed, suggesting good colour durability under the washing conditions used. Fastness ratings are summarised in Table 3: both dyed and ternary fabrics reach Grade 4–5 in washing fastness and dry rubbing fastness. Wet rubbing fastness improves from Grade 3–4 (dyed) to Grade 4 (ternary). This improvement may be associated with the presence of surface-bound protein, which can reduce dye transfer during wet friction.

Figure 6b summarises mechanical performance and moisture transport. The ternary-treated fabric retains approximately 86% of the tensile strength of pristine cotton, indicating that the CPB-based treatment does not cause substantial damage to the fibre matrix. The wicking height of the ternary fabric remains close to that of the control, although it is slightly lower than that of the dyed sample. Such a modest decrease may be related to the partial coverage of surface micropores and/or small changes in surface topography after enzyme incorporation. Overall, capillary transport remains high.

XRD was included here as a structural check rather than as direct evidence of enzyme immobilisation. Since the CPB process involves alkaline dye fixation followed by lysozyme introduction, it was necessary to confirm that these treatments did not visibly disturb the crystalline structure of cotton cellulose. As shown in Figure 6c, the control, dyed, and ternary samples all retain the typical Cellulose I reflections at 2θ ≈ 14.8°, 16.4°, and 22.7°, corresponding to the (1–10), (110), and (200) planes. No clear peak shift or new diffraction feature is observed after dyeing or ternary treatment. This indicates that the CPB-based modification did not cause a detectable change in the main crystalline framework of cotton. The result is also consistent with the tensile strength data, which show that the treated fabric retained most of its original mechanical integrity.

The laundering durability of antibacterial functionality is shown in Figure 6d. Both the antibacterial rate and the ELISA-equivalent lysozyme level decrease gradually with increasing washing cycles yet remain appreciable after multiple washes. The antibacterial trend follows the lysozyme signal, supporting that the observed inhibition is linked to retained lysozyme on the fabric rather than dyeing-related effects and demonstrating the robustness of the ternary-treated cotton under laundering.

Enzyme immobilisation on cellulose is often achieved either by covalent coupling after surface activation (effective but reagent- and step-intensive) or by adsorption/entrapment (simple but typically less wash-resistant). Here we instead work within a reactive dyeing workflow and use RR195 (MCT/VS) as an interfacial mediator so that process sequencing becomes the main control knob for balancing dye fixation and enzyme preservation. The Strategy II vs. Strategy III contrast provides the practical benchmark: introducing lysozyme when reactive motifs are proposed to remain accessible (Strategy II) yields a higher ELISA-equivalent releasable level (52.8 U/L) than post-fixation introduction after thorough rinsing/neutralisation (Strategy III; 39.5 U/L, operational non-reactive control). Alongside this sequence dependence, the retention of antibacterial function after ISO 105-C06 laundering supports the application-level durability of the approach.

## 4. Conclusions

This study presents a CPB-compatible route for introducing lysozyme onto cotton fabric using C.I. Reactive Red 195 (RR195) as both a colourant and an interfacial mediator. Under the optimised conditions identified by the L9 orthogonal design (30 g/L alkali, 6 h batching, 20 g/L dye, and 4 g/L lysozyme), the system gave an ELISA-equivalent releasable lysozyme level of 53.8 U/L together with moderate colour strength (K/S = 6.5).

The mechanistic results support the view that RR195 can provide an electronically activated reactive domain relevant to dye-mediated interfacial formation. The DFT analysis identified the main electrophilic character in the MCT/VS reactive region, which is consistent with the proposed role of the dye in promoting subsequent interaction with lysozyme nucleophiles after fixation on cotton. In the route comparison experiments, sequential impregnation gave the highest ELISA-equivalent level (52.8 U/L), higher than both one-bath and post-fixation introduction. This result indicates that process sequence is important for balancing dye fixation, the retention of reactive character at the fibre surface, and enzyme stability.

The ternary-treated fabric showed strong antibacterial activity, with >99.9% inhibition against *S. aureus* and 96.5% against *E. coli*, and retained substantial antibacterial performance after five washing cycles. At the same time, the treated fabric preserved acceptable textile properties, including about 86% tensile strength retention, favourable moisture transport, and colour fastness of at least Grade 4. These results show that the dye-mediated CPB route can generate a durable bioactive cotton surface without introducing an additional crosslinker or a separate post-curing step.

Overall, the present strategy provides a practical way to construct lysozyme-associated functional interfaces on cellulose within an established reactive dyeing workflow. Although the current evidence does not by itself prove a unique bonding pathway at the fibre surface, the combined DFT, route comparison, surface characterisation, and laundering results support the feasibility of RR195-mediated interfacial immobilisation under CPB conditions. This approach may also be of interest for other cellulose-based substrates where reactive dye chemistry can be used to build durable functional surfaces. Abrasion resistance and pilling behaviour were not examined here, and these wear-related properties should be considered in future application-oriented work.

## Figures and Tables

**Table 1:** L9 (3^4^) orthogonal design for CPB dye-mediated lysozyme immobilisation and corresponding responses.

Run	A: Alkali (g/L)	B: Time (h)	C: Dye (g/L)	D: LZM (g/L)	ELISA (U/L)	Antibacterial Rate (%) *	K/S
1	20 (1)	6 (1)	10 (1)	1 (1)	22.4	65.2	3.1
2	20 (1)	10 (2)	20 (2)	2 (2)	36.5	82.4	5.6
3	20 (1)	14 (3)	30 (3)	4 (3)	41.2	89.6	6.8
4	30 (2)	6 (1)	20 (2)	4 (3)	53.8	99.9	6.5
5	30 (2)	10 (2)	30 (3)	1 (1)	29.7	78.3	8.9
6	30 (2)	14 (3)	10 (1)	2 (2)	38.4	88.5	3.6
7	40 (3)	6 (1)	30 (3)	2 (2)	30.1	75.2	8.4
8	40 (3)	10 (2)	10 (1)	4 (3)	35.6	81.4	3.5
9	40 (3)	14 (3)	20 (2)	1 (1)	15.3	42.1	6.1

*: The antibacterial rate was calculated from CFU counting results against S. aureus, as described in Section 2.6. Numbers in parentheses indicate the factor levels used in the L9 (3^4^) orthogonal design.

**Table 2:** Range analysis (K values and R) for releasable ELISA level, antibacterial response, and colour strength.

Factor	ELISA K1	ELISA K2	ELISA K3	R (ELISA)	Antibacterial K1	Antibacterial K2	Antibacterial K3	R (Anti.)	K/S K1	K/S K2	K/S K3	R (K/S)
A(Akali)	33.4	40.6	27.0	13.6	79.1	88.9	66.2	22.7	5.17	7.67	6.00	1.17
B (Time)	35.4	33.9	31.6	3.8	80.1	80.7	73.4	7.3	6.00	6.00	6.83	0.50
C (Dye)	32.1	35.2	33.7	3.1	78.4	74.8	81.0	6.2	3.40	6.03	7.73	4.63
D (LZM)	22.5	35.0	43.5	21.0	61.9	82.0	90.3	28.4	6.03	5.87	6.00	0.43

**Table 3:** Colour fastness of Reactive Red 195 dyed cotton with and without lysozyme immobilisation.

Sample	Washing (Change)	Washing (Staining)	Rubbing (Dry)	Rubbing (Wet)
Dyed Control	4–5	4–5	4–5	3–4
Ternary System	4–5	4–5	4–5	4

## Data Availability

The original contributions presented in this study are included in the article/Supplementary Material. Further inquiries can be directed to the corresponding authors.

## Supplementary Materials

The following supporting information can be downloaded at https://www.mdpi.com/article/10.3390/polym18101205/s1.
