# Supplementary material for: Enzyme Immobilisation on Cellulose via Bifunctional Reactive Dyes: A Simple Route to Textile-Based Biocatalysts
Source: Polymers (Basel). 2026 May 15;18(10):1205. doi: 10.3390/polym18101205 (PMC13210665; doi:10.3390/polym18101205)
Supplement: Supplementary file 1 [file polymers-18-01205-s001.zip › polymers-4300682-supplementary.pdf]

Enzyme Immobilisation on Cellulose via Bifunctional Reactive Dyes: A Simple Route to Textile-Based Biocatalysts

Guigang Shi [a], Yuhui LI [a], Wenlong Li [a], Ruoying Zhu [ab*], Ying Sun [ab*]

[a]School of Textile Science and Engineering, Tiangong University, Tianjin 300387, China

[b]Key Laboratory of Advanced Textile Composites, Ministry of Education, Tiangong University, Tianjin 300387, China

* Corresponding author. School of Textile Science and Engineering, Tiangong University, Tianjin 300387, China

E-mail address: ruoyingzhu@ tiangong.edu.cn

S1. Antibacterial testing protocol (co-culture method)

Bacterial strains used in the antibacterial assay were Escherichia coli (ATCC 25922) and Staphylococcus aureus (ATCC 6538). Nutrient broth (NB) and nutrient agar (NA) media were used for bacterial culture and plating, respectively. Phosphate-buffered saline (PBS, 0.02 mol/L, pH 7.4) was prepared with $NaH_2PO4\cdot 2H_2O$ (0.6 g), $Na_2HPO_4\cdot 12H_2O$ (5.8 g), and NaCl (9.0 g) in 1 L of distilled water and sterilized at 121 °C for 15 min.

Bacterial working suspensions were prepared by centrifuging activated cultures at 4000 rpm for 10 min, discarding the supernatant, and resuspending the pellets in sterile PBS to obtain ~10^8 CFU/mL. Fabric samples were sterilized by UV irradiation for 2 h. For antibacterial testing, the activated bacterial culture was inoculated (1%, v/v) into freshly prepared NB medium, and sterilized fabric samples were added. The mixture was incubated at 37 °C and 180 rpm overnight. After incubation, aliquots were serially diluted, spread onto NA plates, and incubated at 37 °C for 24 h. Colonies were counted, and inhibition rate was calculated as (N_control − N_sample)/N_control × 100%.

S2. Lysozyme ELISA-equivalent quantification (ELISA)

Lysozyme in the extracted solutions was quantified using a commercial ELISA kit. OD values at 450 nm were recorded on a microplate reader (Tecan Infinite F50). The standard curve was fitted by linear regression: OD = 0.004429 × C + 0.04192, $R^2 = 0.9995$, where C is the ELISA-equivalent lysozyme level (U/L).

Table S1. Standard solutions used for the ELISA calibration.

Standard (U/L)	OD (450 nm)
0.0000	0.0545
25.0000	0.1404
50.0000	0.2815
100.0000	0.4725
200.0000	0.9124
400.0000	1.8229

Figure S1. Standard curve for ELISA assay (OD450 vs ELISA-equivalent lysozyme level).

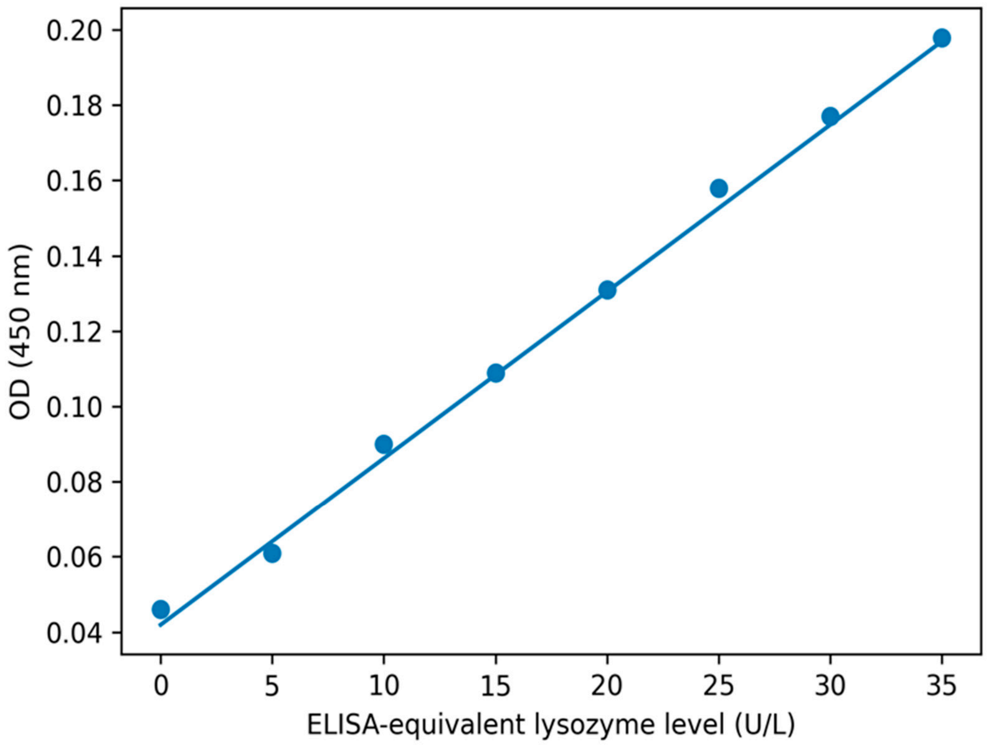

Table S2. Per-replicate ELISA-equivalent lysozyme levels of cotton fabrics prepared via the three introduction strategies (n = 3). Values are reported after the 5-fold sample dilution correction described in Section 2.5 of the main text, and correspond to the means shown in Figure 2a.

Sample ID	Experimental Condition	Replicate	ELISA-equivalent level (U/L)[a]
1	Point 2 (Sequential Impregnation)	1	50.16
2	Point 2 (Sequential Impregnation)	2	53.59
3	Point 2 (Sequential Impregnation)	3	54.65
4	Point 3 (Post-fixation Introduction)	1	37.52
5	Point 3 (Post-fixation Introduction)	2	40.09
6	Point 3 (Post-fixation Introduction)	3	40.88
10	Point 1 (Simultaneous Introduction)	1	39.14
11	Point 1 (Simultaneous Introduction)	2	41.82
12	Point 1 (Simultaneous Introduction)	3	42.64
Control	Untreated Cotton	—	0

[a] Calculated from the ELISA standard curve (OD = 0.004429 × C + 0.04192) and corrected for the 5-fold sample dilution applied during the assay. Raw absorbance data are available from the authors on reasonable request.

S3. Antibacterial performance screening

Table S3. Screening of antibacterial efficacy for different preparation strategies under high bacterial challenge (10^8 CFU/mL).

Note: This preliminary screening was carried out under a higher bacterial challenge (10^8 CFU/mL) than the standard assay reported in the main text (10^6–10^7 CFU/mL, Section 2.6.1), so absolute inhibition values shown here are lower than those in Figure 5. The screening was used only to rank the three introduction strategies and identify the most effective route (Strategy II, Point 2), which was then carried forward into dosage optimisation in the main text.

Group ID	Corresponding Strategy	E. coli Inhibition (%)	S. aureus Inhibition (%)
Control	Pristine Cotton	0	0
1	Point 1 (Simultaneous)	19.9	16.8
2	Point 3 (Post-fixation)	29.8	14.6
3	Point 2 (Sequential)	48.6	40.7

S4. Computational details and Fukui function analysis

S4.1 Computational Methodology

All Density Functional Theory (DFT) calculations were performed using the Gaussian 16 (Rev. C.01) software package1. The molecular structures of the reactive dye (Model 1), cellulose fragment (Model 2, cellobiose), and lysozyme surface residue (Model 3, peptide model) were optimized at the B3LYP/6-311+G(d,p) level with D3BJ dispersion correction. Vibrational frequency analysis confirmed that all optimized structures correspond to local minima (no imaginary frequencies). Electronic properties and condensed Fukui functions were evaluated based on Hirshfeld population analysis using Multiwfn software. The condensed Fukui functions (f^+ for nucleophilic attack and f^- for electrophilic attack) were calculated as:

$$f_k^+ = q_k(N+1) - q_k(N)$$

$$f_k^- = q_k(N) - q_k(N-1)$$

where q_k is the atomic charge at the corresponding state.

S4.2 Optimized Cartesian Coordinates

The optimized Cartesian coordinates (Å) for the studied models are listed below.

(a) Model 1: C.I. Reactive Red 195 (Dye Linker) (Calculated at B3LYP/6-311+G(d,p) level)

C	10.333098	-1.310134	0.395149
C	8.979280	-1.360930	0.167366
C	-2.489054	-1.912970	0.876037
C	10.949309	-0.051002	0.560027
C	8.177557	-0.179083	0.099014
C	6.785850	-0.174766	-0.142179

C	-4.338024	-1.041386	-0.032921
C	-6.620948	-0.378894	-0.879247
C	-7.962159	-0.776207	-0.775848
C	10.232128	1.120063	0.520972
C	8.835032	1.077390	0.294266
C	6.045959	0.997827	-0.145491
C	0.669917	0.116890	-1.138592
C	-2.342906	-0.044881	-0.332339
C	2.032213	0.266986	-0.896144
C	-6.295007	0.743596	-1.657195
C	6.718264	2.253537	0.038655
C	-11.240768	0.475889	0.092033
C	8.074295	2.273547	0.242207
C	2.506697	1.426259	-0.268059
C	-0.219938	1.134286	-0.710662
C	-8.938180	-0.052098	-1.447984
C	-8.635107	1.058435	-2.234475
C	-12.699850	0.153329	0.360707
C	-7.299481	1.443617	-2.324358
C	1.632079	2.461643	0.086574
C	0.270467	2.308673	-0.138117
N	-3.788878	-2.035548	0.713516
N	-5.684197	-1.160653	-0.187966
S	12.708749	0.009235	0.852442
N	0.178761	-0.990599	-1.841099
N	-1.691648	-0.959217	0.409200
N	4.674819	0.769075	-0.370905
N	-3.662608	-0.025819	-0.580862

N	3.868446	1.658839	0.007968
N	-1.612239	0.965824	-0.892325
S	-10.646638	-0.565745	-1.276748
Cl	-1.709746	-3.145946	1.839001

(Note: Hydrogen atoms are omitted for brevity, full set available upon request)

(b) Model 2: Cellulose Fragment (Cellobiose Model)

C	-1.145350	-3.315937	1.763083
C	0.741688	-2.329503	0.289170
C	5.988788	0.016477	-0.336062
C	3.714593	-0.876698	0.127047
C	4.823232	-0.765079	-0.928393
C	-0.718387	-2.233622	0.782271
C	4.061824	1.433347	0.417766
C	1.332389	-0.907013	0.229212
C	5.531892	1.465075	-0.044860
C	0.311076	0.091338	-0.341235
C	-4.305791	-0.801498	0.079504
C	-5.740367	0.840886	-1.166575
C	-4.928771	-0.463449	-1.274416
C	3.706643	2.534701	1.388282
C	-3.900402	1.684148	0.078658
C	-0.919783	0.112023	0.594178
C	-3.306969	0.309664	0.478150
C	-3.554043	2.766931	1.089982
O	5.317726	-2.032903	-1.344806
O	0.804692	-2.885942	-1.018405
O	-2.456293	-3.046484	2.265524
O	7.075449	0.080866	-1.246739

O	2.499261	-0.875412	-0.578835
O	-5.779874	-1.497653	-1.714646
O	5.595180	2.253721	-1.216119
O	3.790574	0.180661	1.062835
O	-0.931438	-0.999394	1.484813
O	-3.737059	-2.089305	-0.063307
O	-5.730837	1.585577	-2.354436
O	0.824490	1.386956	-0.533284
O	-2.066111	0.144815	-0.210762
O	-5.311444	1.610657	-0.045854
O	2.295533	2.482674	1.631338
O	-3.998959	4.057246	0.732647

(c) Model 3: Lysozyme Surface Model (Amino Acid Residues)

C	-2.898280	1.680878	0.325256
C	5.217175	-0.436188	0.443320
C	2.712448	-0.264206	0.086522
C	0.230123	-0.187054	-0.277902
C	-2.101593	0.364338	0.213865
C	-3.878760	-1.465841	-0.052184
C	4.098158	0.032087	-0.477886
C	1.586661	0.381098	-0.735384
C	-2.762593	-0.685944	-0.715630
O	-2.404321	2.743429	0.014429
O	-4.141210	1.579151	0.781599
O	5.058198	-0.800009	1.579945
O	0.091151	-1.377050	0.012268
O	-4.572786	-1.034954	0.851926
O	6.467754	-0.397391	-0.092533

N	-0.783123	0.705461	-0.260470
O	-4.110617	-2.702693	-0.514903
N	1.562024	1.846767	-0.735991

S4.3 Reactivity Descriptors

Table S4. Top electrophilic sites (high f^+) of Reactive Red 195 (Model 1).These nitrogen atoms mark the electron-deficient pocket susceptible to nucleophilic attack.

Index	Atom	f^+	CDD (Dual Descriptor)
44	N	0.0823	0.0806
41	N	0.0748	0.0563
21	C	0.0416	0.0159
6	C	0.0394	0.0147

Table S5. Top nucleophilic sites (high f^-) of Lysozyme Model (Model 3).

These oxygen and nitrogen atoms are identified as nucleophilic sites of the lysozyme model and represent potential interaction centres with the electrophilic region of the dye linker.

Index	Atom	f^-	CDD (Dual Descriptor)
13	O	0.1723	-0.1544
18	N	0.1039	-0.0906
12	O	0.081	-0.0488
15	O	0.0448	-0.0127

S4.4 Visualizations

Figure S2. Atom-numbered structures of the optimized models used for Fukui function analysis: **(a)** Model 1 (Reactive Dye Linker), **(b)** Model 2 (Cellulose Fragment), and **(c)** Model 3 (Lysozyme Surface Model). Note: Atom indices correspond to the data listed in Tables S4 and S5.

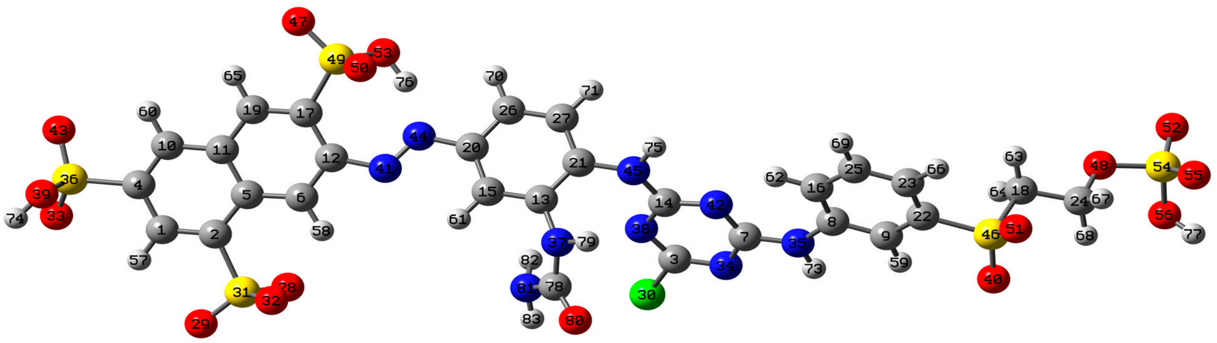

(a) Model 1

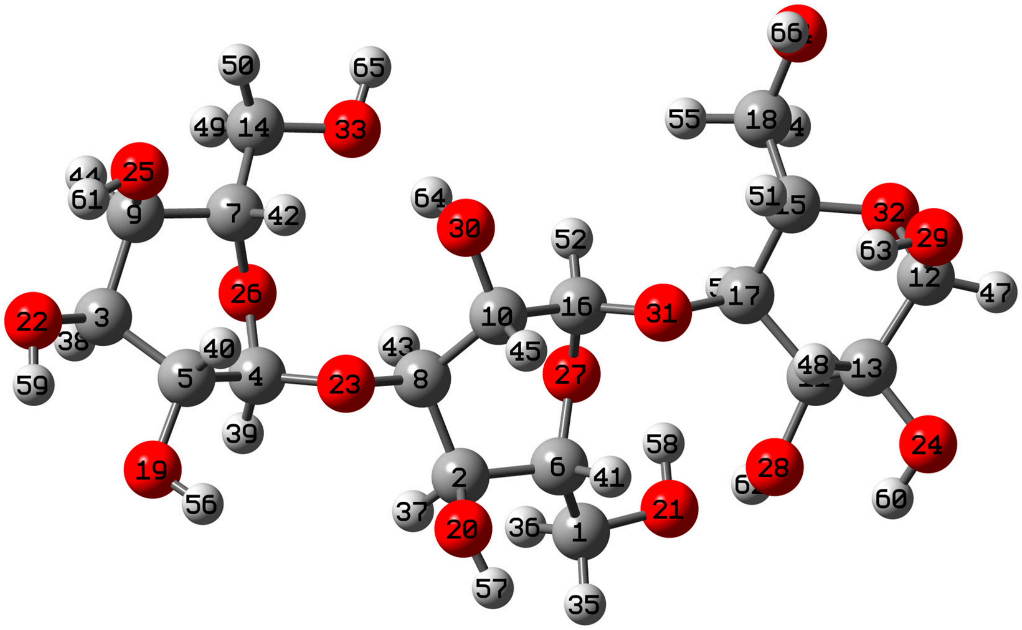

(b) Model 2

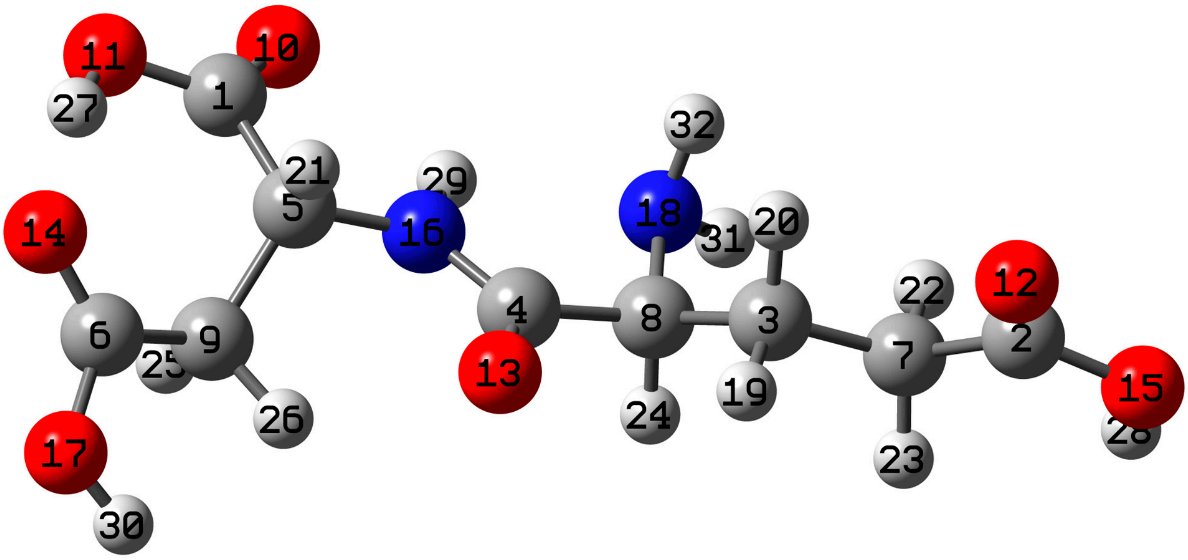

(c) Model 3

Figure S3. Visualization of reactive sites. Isosurface maps of the condensed Fukui functions (f^{+}) for electrophilic attack, f^{-} for nucleophilic attack) and Dual Descriptor (CDD) for (a) Model 1, (b) Model 2, and (c) Model 3. Green regions indicate high susceptibility to reaction.

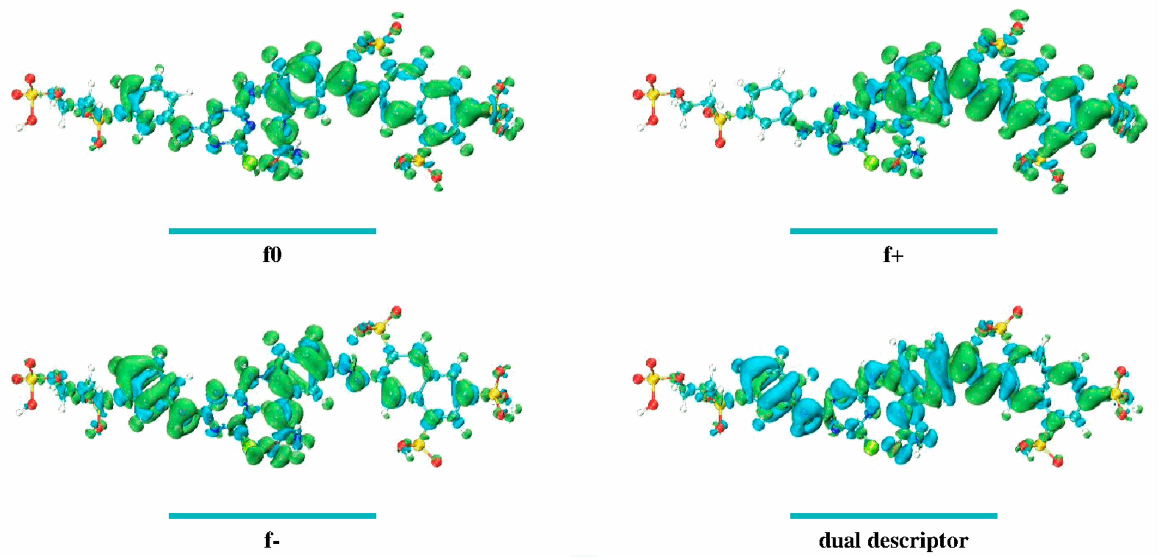

(a) Model 1

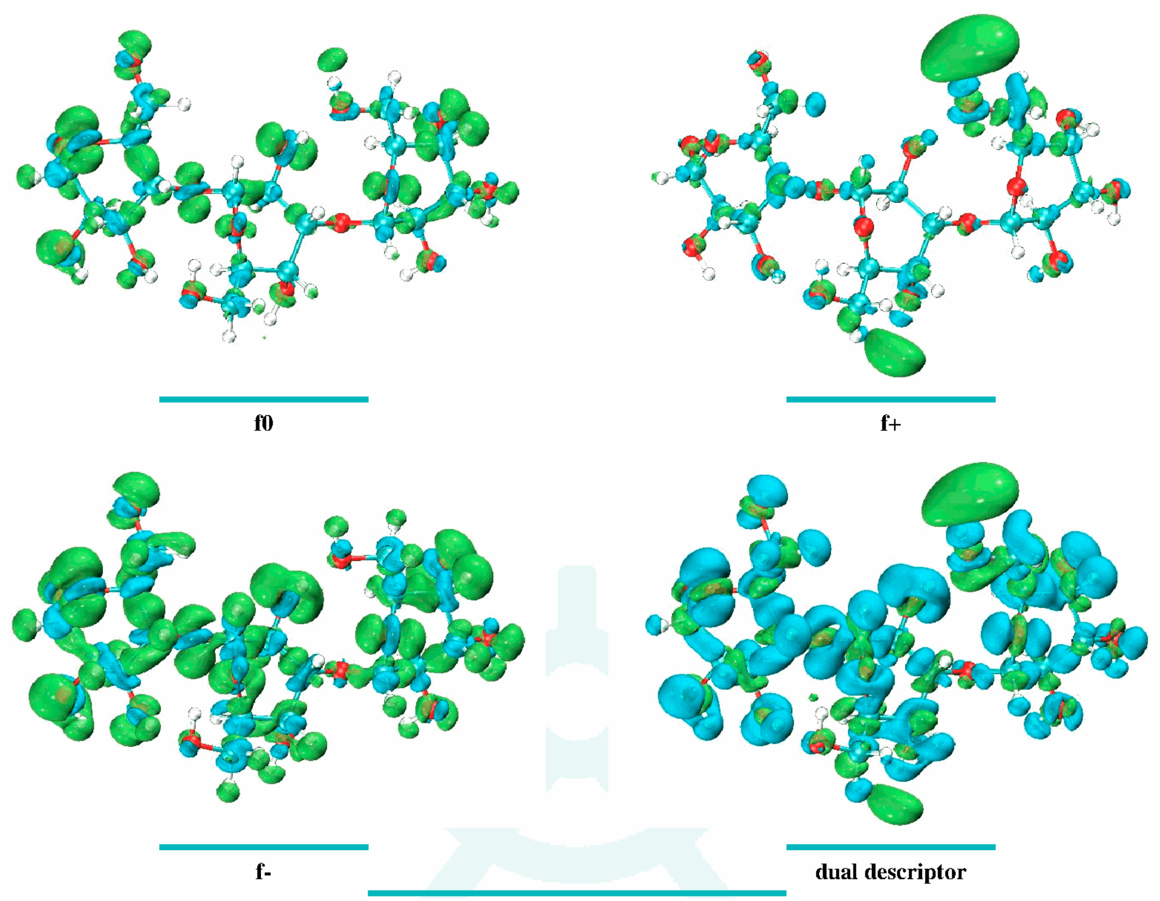

(b) Model 2

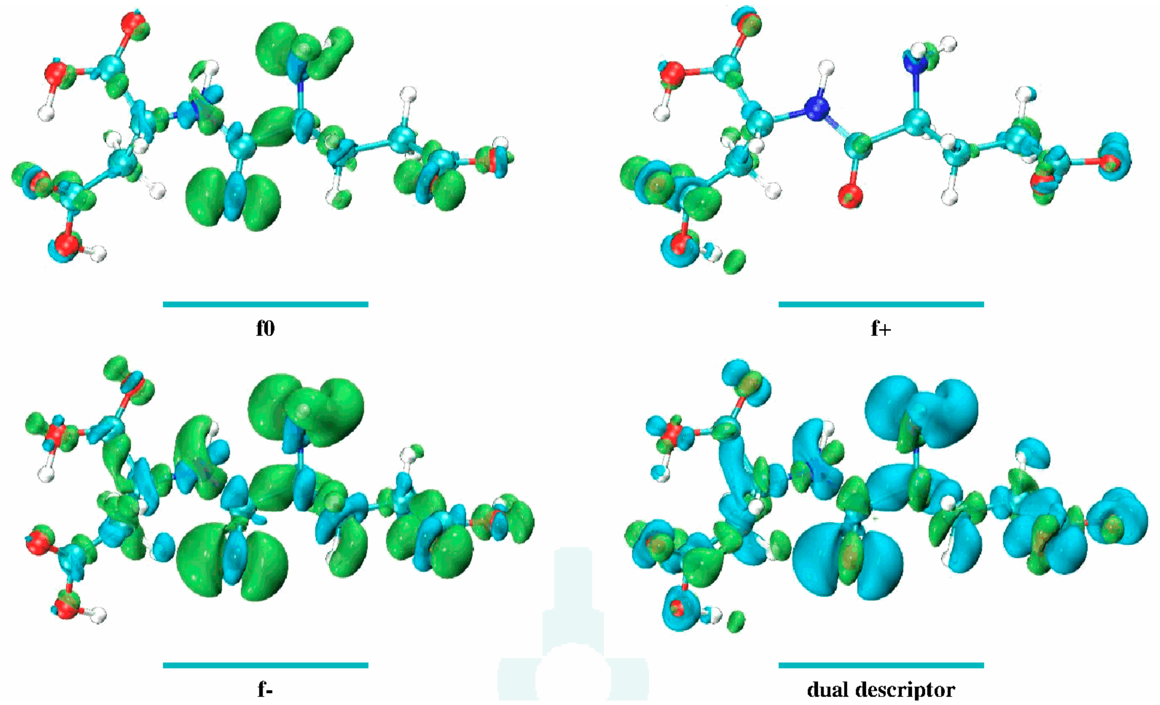

(c) Model 3
